# Computational modeling of therapy on pancreatic cancer in its early stages

**DOI:** 10.1007/s10237-019-01219-0

**Published:** 2019-09-09

**Authors:** Jiao Chen, Daphne Weihs, Fred J. Vermolen

**Affiliations:** 1 Delft Institute of Applied Mathematics, Delft University of Technology, Delft, The Netherlands; 2 Faculty of Biomedical Engineering, Technion-Israel Institute of Technology, Haifa, Israel

**Keywords:** Mathematical modeling, Pancreatic cancer, Cancer therapy, Cell migration, Monte Carlo simulations

## Abstract

More than eighty percent of pancreatic cancer involves ductal adenocarcinoma with an abundant desmoplastic extracellular matrix surrounding the solid tumor entity. This aberrant tumor microenvironment facilitates a strong resistance of pancreatic cancer to medication. Although various therapeutic strategies have been reported to be effective in mice with pancreatic cancer, they still need to be tested quantitatively in wider animal-based experiments before being applied as therapies. To aid the design of experiments, we develop a cell-based mathematical model to describe cancer progression under therapy with a specific application to pancreatic cancer. The displacement of cells is simulated by solving a large system of stochastic differential equations with the Euler–Maruyama method. We consider treatment with the PEGylated drug PEGPH20 that breaks down hyaluronan in desmoplastic stroma followed by administration of the chemotherapy drug gemcitabine to inhibit the proliferation of cancer cells. Modeling the effects of PEGPH20 + gemcitabine concentrations is based on Green’s fundamental solutions of the reaction–diffusion equation. Moreover, Monte Carlo simulations are performed to quantitatively investigate uncertainties in the input parameters as well as predictions for the likelihood of success of cancer therapy. Our simplified model is able to simulate cancer progression and evaluate treatments to inhibit the progression of cancer.

**Electronic supplementary material:**

The online version of this article (10.1007/s10237-019-01219-0) contains supplementary material, which is available to authorized users.

## Introduction

Cancer involves abnormal cellular proliferation, and the disease has the potential to spread to other parts of the body. There are over two hundred different types of cancers including lung cancer, breast cancer, brain cancer, pancreatic cancer, etc. Most cancers have the same progression pattern in the sense that they initiate from a series of gene mutations resulting in uncontrolled proliferation, angiogenesis and metastasis. However, differences exist between cancer types and individuals which necessitate that the treatment be patient-specific, which normally is a combination of surgical resection, radiation therapy and systemic therapy.

The main treatment for pancreatic cancer is surgical resection, yet there is only a small resection rate of 15–20%. In addition, the cumulative 5-year survival rate after the first detection is around 20% (Li et al. 2004) and the median survival is under 6 months. That is because pancreatic cancer is typically diagnosed late, and because of insensitivity to chemotherapy drugs, immune escape and other characteristics. Salmon and Donnadieu (2012) observed that the solid tumors look like islets (T-islets) surrounded by anisotropic desmoplastic extracellular matrix (ECM) that can form a physical barrier to the antitumor immune system response. At the early stage, mutated cancer cells in T-islets trigger massive desmoplasia including a variety of cells and a dense ECM as a natural defense that leads to vascular dysfunction and hence radiotherapy and systemic therapy are significantly hindered. The desmoplasia causes high interstitial fluid pressures that prevent drug diffusion. The high ECM density in pancreatic desmoplasia has been correlated to high concentrations of megadalton glycosaminoglycan hyaluronan (HA) (Provenzano et al. 2012). In pancreatic ductal adenocarcinoma, the HA accumulates in the ECM with a frequency as high as 87% (Shepard 2015). Since the HA can be depleted by the enzyme PEGPH20, a possible therapy could be based on the administering of PEGPH20 with a gemcitabine drug for pancreatic cancer therapy. Jacobetz et al. (2012) showed that combined therapy of PEGPH20 and gemcitabine inhibits tumor growth and improves survival of mice. Moreover, Provenzano et al. (2012) experimentally demonstrated that PEGPH20 + gemcitabine alters tumor biology and increases immune response as well as overall survival in mice. Nevertheless, extended testing is necessary before the combined therapy can be used or even tested in clinical practice.

Systemic toxicity, as a side effect of drug therapy, influences organs and normal tissues. Gemcitabine is used as the frontline drug for the treatment of non-small cell lung cancer and pancreatic cancer; however, gemcitabine is toxic and known to sometimes induce myelosuppression, liver dysfunction, nephrotoxicity, etc. Moreover, cancer recurrence and drug resistance of cancer cells have bottlenecked recurrent or long-term chemotherapy. Therefore, usage of drugs has to be researched and tested massively on animals and even patients. While animal-based experiments have benefited drug development, there are many ethical concerns and preclinical drug restrictions when carrying out these experiments. Mathematical modeling combined with well-designed experiments provides an avenue for cancer therapy research that will allow reduction in the number of animal experiments.

Mathematical modeling enables us to reshape our view of cancer from different perspectives. The process of establishing a mathematical model briefly includes the following steps: choice of a real problem, simplification of a biological phenomenon, establishment of mathematical quantification and performance of numerical simulations. Compared with animal-based experiments, a prominent advantage of mathematical modeling offers an ethical, fast and cost-effective way to test various drug combination strategies, as well as various assumptions and predictions for cancer therapy.

Computational modeling has been developed for a broad spectrum of scales ranging from a few atoms to tissue level with applications to various stages of cancer progression. As early as 1981, Moolgavkar and Knudson (1981) developed a model for carcinogenesis at a cellular level. Similarly, Beerenwinkel et al. (2007) developed a model to explore cancer initiation, in particular the genetic progression with an application to colorectal cancer was considered. Regarding the larger scales, the possibilities to simulate the effects of radiotherapy and chemotherapy for brain tumors by using mathematical modeling were studied by Powathil et al. (2007). Other mathematical models on cancer therapy can be found in Couzin-Frankel (2013) and Namazi et al. (2015). In terms of mathematical modeling related to pancreatic cancer therapy, resources like Louzoun et al. (2014) and Haeno et al. (2012) are rare, and therefore, we develop a computational model to investigate therapeutic combinational possibilities using a Bayesian parameter sensitivity analysis (Campillo-Funollet et al. 2019).

This paper describes a mathematical model that is a continuation of Chen et al. (2018b). The innovations with respect to the aforementioned work are the following: (1) the model has been extended to the simulation of administering drugs that inhibit the proliferation of cancer cells and decay the densely packed, circumferentially oriented ECM around the cancer region; (2) an uncertainty quantification has been carried on the basis of the model parameters to predict the likelihood for successful therapy or further development of cancer. We expect that these principles can be transferred to cancers of different nature. We first consider the injection of enzyme PEGPH20 to degrade HA in the desmoplastic ECM such that the T-lymphocyte infiltration is increased. We then study the effects of subsequent gemcitabine injection to inhibit the proliferation and growth of cancer cells. Injections of both enzyme and drug are modeled by using Green’s functions as solutions of reaction–diffusion equations. Furthermore, the sensitivity of the model with respect to various input parameters is investigated using Monte Carlo simulations.

## Methods

In this paper, we develop the mathematical formalism that is used in the current study. We present the way that the various cell types are modeled, in terms of migration, cell death, proliferation and mutation. Next to the various cell types, we explain how the treatments are incorporated in the model.

### Motivation from experimental observations

**Fig. 1:**
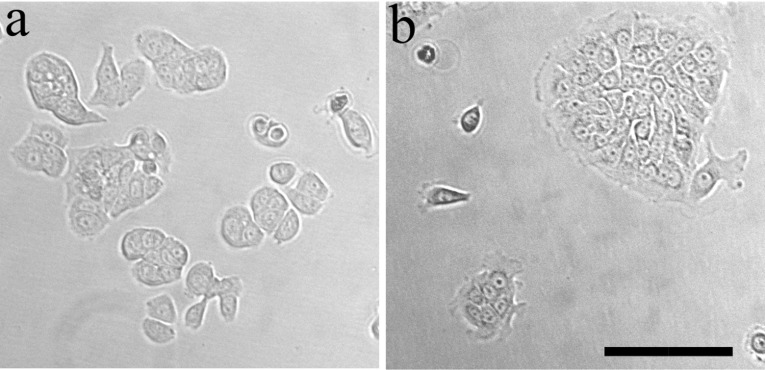
Pancreatic cancer cells on plastic tissue culture dishes. **a** High metastatic potential cell line (AsPC-1). **b** Low metastatic potential or locally invasive cell line (BxPC-3). The periphery of the cluster is structured differently than its interior. Scale bar is 100mm

*Cell culture* Regarding Fig. 1a, b, we have used two commercially available human, pancreatic cell lines (ATCC, Manassas, VA): BxPC-3 (collected from primary site with no evidence for metastasis) and AsPc1 (from metastatic site, ascites). Cells were cultured in their appropriate media as recommended by manufacturer. RPMI-1640 Medium (Biological Industries, Kibbutz Beit Haemek, Israel) supplemented with 10 vol% FBS (ThermoFisher Scientific, Waltham, MA), 1 vol% of penicillin-streptomycin (Biological Industries, Kibbutz Beit Haemek, Israel), 0.46 vol% D-Glucose solution, 1 vol% HEPES solution and 0.66 vol% sodium bicarbonate solution (all from Sigma, St Louis, MO). Cells were maintained in a sterile incubator at 37 $^{\circ }\hbox {C}$, 5% CO_2_ and high humidity. Cells were frozen at low passages from ATCC stock (i.e., 3–5), and for experiments cells were thawed and used in passages 7–30 from the ATCC stock.

*Microscopy and imaging* Cells seeded on 10-*cm* tissue culture plastic plates were imaged using an inverted, epifluorescence Olympus IX81 microscope, with a 20x/0.5NA differential interference contrast (DIC, Nomarski optics) air-immersion, objective lens. Cells at random locations were imaged while being maintained in $37\,^{\circ }\hbox {C}$ , 5% CO_2_, and high humidity (90%), in an on-stage and an on-microscope incubator (Life Imaging Services, Switzerland), to sustain their viability for prolonged periods of time.

*Assumptions* Many of the fundamental biological assumptions in the current model are taken from Chen et al. (2018b), since the current paper is an extension of Chen et al. (2018b) where therapy is taken into account. We summarize the biological assumptions, which are needed to have a tractable model.

1. We only consider three phenotypes: epithelial cells, cancer cells and T-lymphocytes;
2. Each cell can be in the following two states: dead or viable;
3. Currently, we consider a two-dimensional domain of computation to avoid very large computation times. Further, cell deformation is not taken into account for reasons of computational efficiency, and therefore, all cells are assumed to be circular;
4. Because of the lack of information regarding the composition of the desmoplastic stroma, we assume its density to be uniform. We do take into account the variability of the orientation of the desmoplastic stroma by using the orientation tensor;
5. According to the experimental studies by Reinhart-King et al. (2008), cells are able to communicate by mechanical forces exerted on the surrounding substrate. This mode of long-distance communication has been incorporated in the current paper on the basis of the strain energy density. In the modeling, the strain energy density impacts the direction of migration of the cells;
6. Intercellular contact is simulated by modeling the cells as elastic, soft circles in the 2D framework. Here, Hertz contact mechanics has been used, which was also proposed in the mouse experimental paper by Gefen (2010), which treats the invagination of viruses into cells;
7. Cells are subject to various modes of migration. In this paper, we assume that chemotaxis of T-lymphocytes migration results from the secretion of a generic chemokine that is secreted by the cancer cells. Furthermore, since the extracellular matrix always contains inhomogeneities, of which the exact locations are unknown, we incorporate a random component to the migrational vectors of the cells. This randomness is modeled by a random walk, which is a very common approach in the literature (Stokes and Lauffenburger 1991).
8. Cumming et al. (2009) modeled orientation effects of extracellular matrix in the context of wound healing in the skin. Since Salmon and Donnadieu (2012) observed T-lymphocytes peripheral migration around T-islets in cancer, where the cells only exhibit very little movement in the direction perpendicular to the periphery, we follow the approach of Cumming et al. (2009) to incorporate orientational variations in the desmoplastic stroma.
9. According to the experimental studies by Kar et al. (2009), homogeneous cultures of cell exhibit the same cell cycle if it comes to division and death. However, the rates on which the cell cycles proceed differ from cell to cell. Kar et al. (2009) observed a random pattern which they catch in statistical distributions. Therefore, we incorporate cell division, mutation and death as random processes.

**Fig. 2:**
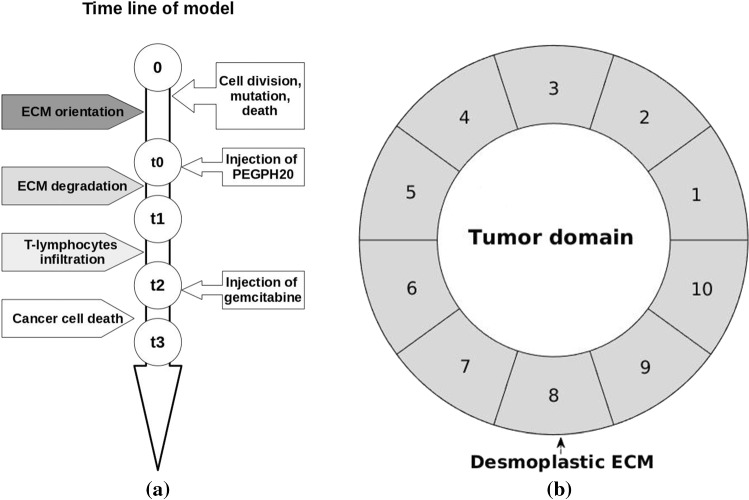
Schematic figures. **a** The timeline of T-islets model in domain $\Omega$. **b** A circular desmoplastic ECM, which is divided into ten subdomains (indexed from 1 to 10) for computation

Next, we incorporate the assumptions behind the therapy, which is based on the administering of the cocktail of PEGPH20 and gemcitabine. This therapy has been tested on mice, which results in an improvement in survival of mice subject to pancreatic cancer. We model the impact of therapy by the use of the following assumptions:

1. We consider a circular domain of computation, which is in line with the pancreatic experimental observations (Olive et al. 2009; Öhlund et al. 2017). Our Fig. 1b also demonstrates this circular domain, where an early circular cluster of densely packed cancer cells is observed with edge cells exhibiting a unique morphology. This is also found in the studies by Salmon and Donnadieu (2012). Therefore, a circular cancer domain with a circumferentially ring-shaped desmoplastic stroma is modeled and depicted in Fig. 2b.
2. Jacobetz et al. (2012) indicated that PEGPH20 can possibly be used to degrade the desmoplastic stroma. Therefore, we assume that PEGPH20 makes the orientation of the desmoplastic stroma more isotropic, and hence, the T-lymphocytes migration into the T-islets is enhanced;
3. Gemcitabine is a very general drug for chemotherapy against pancreatic cancer. This chemical is known to inhibit the DNA synthesis, and hence, cell proliferation is frustrated (Plunkett et al. 1995). Therefore, we assume that gemcitabine suppresses the proliferation of cancer cells;
4. Since it is hard to obtain constitutive relations for the diffusivities of the various chemicals (drugs and cancer cell-secreted chemokine), we assume that diffusion of all chemicals is based on Fick’s law for linear diffusion. Furthermore, we are only interested in the qualitative behaviors of diffusion, and therefore, we use Green’s functions to describe the concentration fields. A further motivation for this approach is that the Green’s functions easily provide explicit relations for concentrations and their gradients, which are needed for modeling chemotaxis, without the need of mapping from finite element meshes (which possibly results into a loss of accuracy).

### Migration of epithelial and cancer cells

Cancer initiates from genetic mutations, and therefore, we consider the normal epithelial cells, which can mutate to cancer cells, and cancerous cells in a bounded computational domain $\Omega \subset {\mathbb{R}}^2$. The set of epithelial and cancerous cells at time *t* is denoted by ${\mathbb{W}}(t)$. Cells migrate in the domain $\Omega$ and interact with each other as well as with its microenvironment, e.g., substrate in 2D or ECM in 3D. Generally, cell migration is classified into *amoeboid* or mesenchymal movement. Cancer cells have the ability to change state between these two migrational modes in order to adapt to environmental changes. In the current work, we assume that cells migrate according to mechanical signals as a result of substrate deformation caused by neighbor cells’ adhesion and traction (Massalha and Weihs 2017). For completeness, we present some of the equations from Vermolen and Gefen (2012). Slight deformation of substrate gives a strain energy *U* as

1 $$\begin{aligned} U = \frac{{1}}{2} V E \varepsilon ^2, \end{aligned}$$

where *V* and *E* denote the deformation volume and Young’s modulus. Note that $\varepsilon$ defines strain of the substrate given by $\varepsilon = \frac{{d}}{L}$ with *d* in deformed vertical displacement and *L* in thickness of the substrate. Then, the strain energy density (total energy per unit of volume) $M_i^{\mathrm{0}}$ is calculated by

2 $$\begin{aligned} M_i^{\mathrm{0}}= \frac{{1}}{2} E_{\mathrm{s}}(\mathbf{r}_i) \varepsilon ^2, \quad \mathrm{for} \ i \in {\mathbb{W}}(t). \end{aligned}$$

Here, $E_{\mathrm{s}}(\mathbf{r}_i)$ denotes the Young’s modulus of substrate at the center of cell *i* and $\mathbf{r}_i = (x_i, y_i)$ is its corresponding position. If Eq. (2) is combined with Hooke’s Law $\varepsilon = \frac{{1}}{E_{\mathrm{s}}(\mathbf{r}_i)}\frac{{F_i}}{\pi R^2}$, then we get

3 $$\begin{aligned} M_i^{\mathrm{0}}= \frac{{1}}{2\pi ^2}\frac{{F_i^2}}{E_{\mathrm{s}}(\mathbf{r}_i)R^4}, \quad \mathrm{for} \ i \in {\mathbb{W}}(t). \end{aligned}$$

For cell *i* with radius *R*, $F_i$ represents the exerted force on the substrate. The total strain energy density that a cell detects originates from itself as well as from the other neighboring cells. Cells are able to detect signals from other cells if a certain threshold is exceeded for the strain energy density (Chen et al. 2018b; Reinhart-King et al. 2008). Since the mechanical signal decays with the distance, we compute the attenuation of the signal from another cell *j* by

4 $$\begin{aligned} M_i(\mathbf{r}_j)= M_i^{\mathrm{0}}\mathrm{exp}\left\{ -\lambda _i \frac{{\parallel \mathbf{r}}_i- \mathbf{r}_j \parallel }{R}\right\} , \quad \mathrm{for} \ i, j \in {\mathbb{W}}(t). \end{aligned}$$

The attenuation factor $\lambda _i$ can be approximated by $\lambda _i=\frac{{E_{s}}(\mathbf{r}_{i})}{E_c}$ (Merkel et al. 2007), where $E_c$ is the Young’s modulus of the cell. Since the strain energy density is a scalar, the total value of one cell at position $\mathbf{r}_i$ can be obtained by summing, that is

5 $$\begin{aligned} \begin{aligned} M(\mathbf{r}_i)&= \sum _{j \in {\mathbb{W}}(t)} M_j(\mathbf{r}_i) \\&= M_i^{\mathrm{0}}+ \sum _{i,j\in {\mathbb{W}}(t)_{j \ne i}}M_{j}^{0}\exp \left\{ -\lambda _{j} \frac{{\parallel \mathbf{r}}_{i}-\mathbf{r}_{j}\parallel }{R}\right\} . \end{aligned} \end{aligned}$$

Based on the work by Vermolen and Gefen (2012), the displacement direction of a cell is determined by the unit vector between itself and other cells, for example, $\mathbf{e}_{ij} = \frac{{\mathbf{r}}_{i}-\mathbf{r}_{j}}{\parallel \mathbf{r}_{i}-\mathbf{r}_{j}\parallel }$ for cell *i* and cell *j*. At time *t*, the final displacement direction $\mathbf{z}_{i}$ of cell *i* can be obtained by the following linear combination of unit vectors obtained through the interconnection vectors between the cells,

6 $$\begin{aligned} \mathbf{z}_{i}=\sum _{j=1_{j\ne i}}^{n}M_{j}(\mathbf{r}_{i}(t))\mathbf{e}_{ij}, \quad \mathrm{for} \ i \in {\mathbb{W}}(t). \end{aligned}$$

Under the mechanical stimulus, the total displacement of a cell per time step $\mathrm{d}t$ is given by

7 $$\begin{aligned} \mathrm{d}{} \mathbf{r}_{i}(t)=\alpha _{i} M(\mathbf{r}_{i}(t))\hat{\mathbf{z}}_{i}\mathrm{d}t, \quad \mathrm{for} \ i \in {\mathbb{W}}(t). \end{aligned}$$

In Eq. (7), $\hat{\mathbf{z}}_{i}$ is a unit vector ($\hat{ \mathbf{z}}_{i}=\frac{{\mathbf{z}}_{i}}{\parallel \mathbf{z}_{i}\parallel }$) and the velocity parameter $\alpha _i$ follows from Gefen (2010) and is given by

8 $$\begin{aligned} \alpha _{i}= \frac{{\beta _{i}}R^3}{\mu F_{i}}, \quad \mathrm{for} \ i \in {\mathbb{W}}(t), \end{aligned}$$

where $\mu$ is the cell-substrate friction coefficient and $\beta _i$ represents the mobility coefficient of the area of one cell that is in contact with the substrate.

Cell contact inhibition is a biological mechanism to inhibit cell proliferation and to decrease mobility. As a result, the migration speed can be dampened if two cells collide. Therefore, we incorporate a repulsive invagination force $M^{ij}$ between cell *i* and cell *j* as introduced in Gefen (2010), which increases with the impinging distance. The equation reads as

9 $$\begin{aligned} M^{ij}=\frac{{4}}{15 \sqrt{2}}\frac{{E_c}}{\pi }\left( \frac{{h}}{R}\right) ^{\frac{{5}}{2}},\quad \mathrm{for} \ i, j \in {\mathbb{W}}(t). \end{aligned}$$

The variable *h* is the distance of impingement given by $h = \mathrm{max}(2R-\parallel \mathbf{r}_{ij}\parallel , 0)$, where $\mathbf{r}_{ij}$ defines the distance between cell *i* and cell *j*. Note that this equation guarantees that any number of cells will not overlap too much during collision.

Taking the unpredictability of cell migration into account, we extend the model with a temporal stochastic process in the form of a Wiener process ($W \sim {{\mathcal {N}}}(0, \mathrm{d}t)$). In summary, the displacement of epithelial and cancer cells is determined by the strain energy density, total repulsive force $M^{\mathrm{mc}}(\mathbf{r}_i)$ and random walk, and thereby, the revised equation is written as

10 $$\begin{aligned} \mathrm{d}{} \mathbf{r}_i(t)= \alpha _i {\hat{M}}_i(\mathbf{r}){\hat{\mathbf{z}}_{i}} \mathrm{d}t+\eta \mathrm{d}{} \mathbf{W}(t), \quad \mathrm{for} \ i \in {\mathbb{W}}(t), \end{aligned}$$

where ${\hat{M}}_i(\mathbf{r})$ is the total mechanical signal, which is given by ${\hat{M}}_i(\mathbf{r}) = M(\mathbf{r}_{i})-M^{\mathrm{mc}}(\mathbf{r}_i)$ and $\eta$ represents a constant in this random walk. Further, $\mathrm{d}{} \mathbf{W}(t)$ represents a vector with independent samples from ${{\mathcal {N}}}(0, \mathrm{d}t)$. In ${\mathbb{R}}^2$ and ${\mathbb{R}}^3$, the number of components of $\mathrm{d}{} \mathbf{W}(t)$ is two and three, respectively. To solve the problem, we use the Euler–Maruyama method (Kloeden and Platen 2013), which boils down to the ordinary forward Euler method combined with the Wiener Process:

11 $$\begin{aligned} \mathbf{r}_i^n = \mathbf{r}_i^{n-1} + \Delta t \alpha _i {\hat{M}}_i(\mathbf{r}^n) + \eta \Delta \mathbf{W}(t),\quad \mathrm{for} \ i \in {\mathbb{W}}(t). \end{aligned}$$

Here, $\Delta \mathbf{W}(t)$ represents a vector with independent samples from ${{\mathcal {N}}}(0, \Delta t)$. The above equation contains a time integration in which a part is random from the Wiener process. Using a higher-order method makes the numerical error smaller than the actual uncertainty. Therefore, we decided to use the ordinary Euler–Maruyama method in which the deterministic part is treated by a first-order forward Euler method. However, to ensure the numerical stability, the time step cannot be chosen arbitrarily large. If we restrict the displacement of a cell step to one-fourth of the cell diameter, then the time step is bounded by ${\Delta } t \le \frac{{R}}{2 {\rm max}\parallel \mathbf{v}_i\parallel }$ with $\mathbf{v}_i$ denoting an equilibrium velocity of cell *i*.

### Migration of T-lymphocytes

Migration of cells can be driven by several cues. Such cues can be chemicals, electricity, mechanical properties (such as stress or elasticity) and light. For the locomotion of T-lymphocytes, we take chemotaxis and small range impingement into account. According to Van Damme et al. (1992) and Kershaw et al. (2002), immune cells like cytotoxic T-lymphocytes move toward the gradient of chemokines secreted by cancerous cells. We use ${\mathbb{K}}(t)$ and ${\mathbb{T}}(t)$ to represent the set of cancer cells and the T-lymphocytes at time *t*, respectively. Each cancer cell is modeled as a point source, and therefore, we consider the Dirac delta distribution $\delta (\mathbf{r})$ to model the chemokine secreted by each cancer cell. Then, the concentration of the chemokine change is described as

12 $$\begin{aligned} \frac{{\partial c}}{\partial t}- D_\mathrm{c}\Delta c = \sum _{j \in {\mathbb{K}}(t)}\gamma _j(t) \delta (\mathbf{r}-\mathbf{r}_j(t)), \ \mathrm{for} \ j \in {\mathbb{K}}(t). \end{aligned}$$

In Eq. (12), *c*, $D_\mathrm{c}$ and $\gamma _j(t)$ denote chemokine concentration, diffusivity and secretion rate by cancer cells at time *t*. For the sake of simplicity and applicability of the Green’s functions and in order to avoid the enlargement of the parameter space in the model, we take all diffusion coefficients constant over the various subdomains in all the simulations. Regarding the time-dependent scheme, it takes computational time and memory to store all the positions of cancer cells at all times. Therefore, we solve the steady-state part of Eq. (12), which results into

13 $$\begin{aligned} \frac{{\partial c}}{\partial x}(x,y)= & {} - \sum _{j\in {\mathbb{K}}(t)}\frac{{\gamma _j(t)}}{2\pi D_\mathrm{c}}\frac{{x - x_j(t)}}{\parallel \mathbf{r}-\mathbf{r}_j(t)\parallel ^2},\nonumber \\ \frac{{\partial c}}{\partial y}(x,y)= & {} - \sum _{j\in {\mathbb{K}}(t)}\frac{{\gamma _j(t)}}{2\pi D_\mathrm{c}}\frac{{y - y_j(t)}}{\parallel \mathbf{r}-\mathbf{r}_j(t)\parallel ^2}. \end{aligned}$$

Analogously, any two T-lymphocytes are not allowed to overlap too much, and thus, the contact inhibition is considered by using mechanical repulsion $M^{\mathrm{mc}}$ in Eq. (9). Furthermore, the random walk is incorporated as well to mimic the unpredictable migratory behaviors of T-lymphocytes. However, the remote mechanical cues are disregarded for the migration of T-lymphocytes. Then, the displacement of T-lymphocytes is written as

14 $$\begin{aligned} \begin{aligned}&\mathrm{d}{} \mathbf{r}_j(t) = \beta \nabla c(t, \mathbf{r}_j(t))\mathrm{d}t + \eta \mathrm{d}{} \mathbf{W}(t)-M^{\mathrm{mc}}(\mathbf{r}_j)\mathbf{z}_j\mathrm{d}t, \\&\quad \mathrm{for} \ j \in {\mathbb{T}}(t), \end{aligned} \end{aligned}$$

where $\beta$ defines the chemotactic constant. Similarly, $\mathrm{d}{} \mathbf{W}(t)$ is a vector Wiener process. The displacement of T-lymphocytes is dealt with by using the same Euler–Maruyama method expressed by

15 $$\begin{aligned} \begin{aligned} \mathbf{r}_j^n =\,&\mathbf{r}_j^{n-1}+ \nabla c(t, \mathbf{x}_j^{n-1})\Delta t + \eta \Delta \mathbf{W} \\&-M^{\mathrm{mc}}(\mathbf{r}_j^{n-1})\mathbf{z}_j^{n-1}\Delta t,\ \mathrm{for}\ j \in {\mathbb{T}}(t). \end{aligned} \end{aligned}$$

For an overview of cross talk between cells and microenvironment, the reader is referred to Fig. 1 in Chen et al. (2018b).

### Stochastic processes: cell division, mutation and death

Cell proliferation, mutation and death are some of the fundamental processes of cells regulated by genes, intracellular interaction and microenvironment. To simplify the model, stochastic processes are considered to simulate the probability of cell division, mutation and death (Vermolen 2015). We hypothesize that the probability of cell division, mutation and death is only influenced by the total strain energy density one cell endures. Then, the probability density for $t > t_n$ is given by

16 $$\begin{aligned} f_{t_n}(\lambda , t) = \lambda \mathrm{exp}(-\lambda (t-t_n)), \quad \mathrm{for} \ t > t_n, \end{aligned}$$

where $\lambda > 0$ is the probability rate of cell division, mutation or death per hour. This probability density is common in modeling waiting times of discrete phenomena, see Grimmett and Stirzaker (2001, p. 95). Hence, the probability is achieved by time integration

17 $$\begin{aligned} \begin{aligned} P(t\in (t_n, t_n+\Delta t))&= \int _{t_n}^{t_n+\Delta t}f_{t_n}(\lambda , t)\mathrm{d}t \\&\simeq 1-\mathrm{exp}(-\lambda \Delta t). \end{aligned} \end{aligned}$$

Note that the incidence of cell division, mutation or death is determined by $\xi$ as

18 $$\begin{aligned} 0 \le \xi \le 1-\mathrm{exp}(-\lambda \Delta t), \end{aligned}$$

where $\xi \sim u[0,1]$ is generated from an uniform distribution. Since most chemotherapy drugs target on DNA generation and thereby inhibit cell division, the probability rate $\lambda$ of cancer cell division and mutation reads as

19 $$\begin{aligned} \lambda = {\left\{ \begin{array}{ll} \lambda _0 \\ \lambda (c(t)) = \lambda _0\mathrm{exp}(-Ac_{\mathrm{drug}}) \end{array}\right. }, \end{aligned}$$

where $\lambda _0$ denotes the initial probability rate and $\lambda (c(t))$ represents the probability rate for cancer cell proliferation under the influence of drug therapy.

### Desmoplastic ECM

Despite the enormous number of cellular studies, the interaction between cancer cells and the microenvironment is still poorly understood. In pancreatic cancer, the components of the desmoplastic ECM around T-islets are likely dynamic, and thus, its function is controversial. Some studies (Salmon and Donnadieu 2012; Hanahan and Weinberg 2011) suggest that the desmoplastic ECM supports cancer progression, whereas some studies hint to the contrary (Rhim et al. 2014). However, there is a consensus that cancer cells in the pancreas are able to reshape the normal ECM to adapt to their survival needs. Some of the properties of the desmoplastic ECM can be generalized as, (1) profuse fibers that are arranged in parallel to the circumference of the T-islets that leads to an anisotropic environment; (2) abundant regeneration of HA results in local, stiff tissue; and (3) the stiff desmoplastic ECM acts as a solid defense that hinders the entry of many agents, e.g., immune cells, blood vessel generation, drugs, etc.

Due to chemotaxis, T-lymphocytes tend to move toward the gradient of the concentration of chemokines secreted by cancer cells (Salmon and Donnadieu 2012). However, their migration is guided by the desmoplastic ECM orientation once T-lymphocytes enter the anisotropic desmoplastic ECM (Bougherara et al. 2015). As a result, T-lymphocytes preferably migrate in the tangential direction and slow down in the radial direction, which results in the behavior that cells are migrating around the tumor, and hence, the cells do not penetrate the tumor. To model the orientation in the two-dimensional framework, we introduce an orientation tensor $\varPsi (t,\mathbf{x})$ (Cumming et al. 2009)

20 $$\begin{aligned} \varPsi (t,\mathbf{x}) = \begin{pmatrix} \varPsi _{xx} &{} \varPsi _{xy} \\ \varPsi _{xy} &{} \varPsi _{yy} \end{pmatrix}. \end{aligned}$$

The tensor is symmetric according to the tangential and radial directions. Thereby, the orientation tensor is calculated by

21 $$\begin{aligned} \varPsi = v^0e^{-ks}\lambda _1 \mathbf{w_1w_1}^T+v^0\lambda _2\mathbf{w_2w_2}^T, \end{aligned}$$

where $\mathbf{w_1}$ and $\mathbf{w_2}$ are orthogonal eigenvectors denoting the radial and tangential components (Cumming et al. 2009). The eigenvalues $\lambda _1$ and $\lambda _2$ are the corresponding weights. Furthermore, there is an attenuation in radial speed with rate constant *k* reading as $\frac{{\partial v}}{\partial s} = -kv$. Here, *s* represents the penetration depth and finally *v* is given by $v = v^0e^{-ks}$ with an initial velocity $v^0$ on the ECM external boundary, see Eq. (21). Finally, the displacement of T-lymphocytes under the influence of collagen orientation is adjusted to

22 $$\begin{aligned} \begin{aligned} \mathbf{r}_j^n =\,&\mathbf{r}_j^{n-1}+ \mu _j \varPsi (\nabla c(t, \mathbf{r}_j^{n-1})\Delta t + \eta \Delta \mathbf{W}) \\&-M^{\mathrm{mc}}(\mathbf{r}_j^{n-1})\mathbf{z}_j^{n-1}\Delta t, \ \mathrm{for} \ j \in {\mathbb{T}}(t), \end{aligned} \end{aligned}$$

where $\mu _j$ denotes the chemotactic mobility rate.

At present, we have the relevant experimental results shown in Fig. 1 to support our simplified model. As above mentioned, pancreatic cancer is typically diagnosed at late stages with a high metastasis risk. Highly metastatic pancreatic cancer cells, already in their invasive state, are more likely to remain as individuals, especially during invasion, see Fig. 1a. In contrast, noninvasive cancer cells accumulate into dense clusters on plates, likely emulating rapid proliferation in the early stages of tumor growth (see Fig. 1b). We consider the structure of the cluster of cancer cells, with its highly dense cells, as already similar to a circular islet, which is surrounded by tangentially oriented desmoplastic ECM.

### Enzyme and drug injection

Pancreatic cancer frustrates the human immune system and builds a physiological barrier to protect itself (Feig et al. 2012; Provenzano et al. 2012). These two properties render chemotherapy pointless, since most chemotherapy drugs are given by intravenous injection and subsequently arrive at the tumor via the blood stream (Neesse et al. 2011). Compared with other types of cancers, the regeneration of new blood vessels does not take place in the anisotropic ECM in pancreatic cancer, whereas cancer cells are capable of surviving under conditions with few nutrients due to insufficient blood supply (Awale et al. 2006). Therefore, in our simulations, we initially mimic a treatment that in the first step aims at the degradation of the ECM that will then allow drug delivery to the cancer cells.

Jacobetz et al. (2012) showed that abundant HA impairs vascular function and hinders drug delivery, and hence, degradation of HA combined with chemotherapy drugs could be an option for treatment. The enzyme PEGPH20 is considered here to degrade HA rapidly and efficiently and is administered by injections. The injection can be regarded as a source point when using the Dirac delta distribution $\delta$ and the corresponding concentration $c_{\mathrm{en}}$ diffuses based on

23 $$\begin{aligned} \frac{{\partial c_{\mathrm{en}}}}{\partial t}- D_{\mathrm{en}}\Delta c_{\mathrm{en}} = \sum _{p \in {\mathbb{P}}(t)}\gamma _{\mathrm{en}}(t) \delta (\mathbf{r}-\mathbf{r}_p(t)), \end{aligned}$$

where $D_{\mathrm{en}}$ and ${\mathbb{P}}(t)$ denote the enzyme diffusivity and the set of multiple injections. The injection rate $\gamma _{\mathrm{en}}(t)$ of each injection site is defined as

24 $$\begin{aligned} \gamma _{\mathrm{en}}(t) = {\left\{ \begin{array}{ll} \gamma _0, \quad \mathrm{if} \ t_0< t \le t_1 \\ 0, \quad \ \mathrm{else} \end{array}\right. }, \end{aligned}$$

which means that PEGPH20 is injected at time $t_0$ until time $t_1$ and no more enzyme is given afterward. A schematic timeline of T-islet model in the domain $\varOmega$ with important marks is shown in Fig. 2a, where the time of drug administration is referred to Jacobetz et al. (2012). When the percentage of cancer cells amount in total cells amount exceeds 35%, the PEGPH20 starts to be injected and time is marked as $t_0$. To simplify the description of the process, we assume that the enzyme is injected once at position $\mathbf{r}_p$ nearby T-islets, and thence, the concentration of enzyme with respect of time *t* at location $\mathbf{r}$ is

25 $$\begin{aligned} \begin{aligned} c_{\mathrm{\mathrm{en}}}(\mathbf{r})&= \int _{0}^{t}\frac{{\gamma _{\mathrm{en}}}(t)}{4\pi D_{\mathrm{en}}(t-s)}e^{\frac{{\parallel \mathbf{r}}-\mathbf{r}_p\parallel ^2}{4D_{\mathrm{en}}(t-s)}}\mathrm{d}s \\&= \int _{t_0}^{t_1}\frac{{\gamma _0}}{4\pi D_{\mathrm{en}}(t-s)}e^{\frac{{\parallel \mathbf{r}}-\mathbf{r}_p\parallel ^2}{4D_{\mathrm{en}}(t-s)}}\mathrm{d}s. \end{aligned} \end{aligned}$$

The second equality sign results after applying Eq. (24). Once the drug has been injected, it diffuses to its surroundings according to

26 $$\begin{aligned} \frac{{\partial c_{\mathrm{drug}}}}{\partial t} - D_{\mathrm{drug}}\Delta c_{\mathrm{drug}} = \sum _{q \in {\mathbb{D}}(t)}\gamma _{\mathrm{drug}}(t) \delta (\mathbf{r}-\mathbf{r}_q(t)), \end{aligned}$$

where $D_{\mathrm{drug}}$ and ${\mathbb{D}}(t)$ denote the drug diffusivity and the set of multiple injections. Subsequently, the injection rate $\gamma _{\mathrm{drug}}$ of chemotherapy drug gemcitabine during time interval $(t_2, t_3)$ as well as afterward is given by

27 $$\begin{aligned} \gamma _{\mathrm{drug}}(t) = {\left\{ \begin{array}{ll} \gamma _0, \quad \mathrm{if} \ t_2< t \le t_3 \\ 0, \quad \ \mathrm{else} \end{array}\right. }, \end{aligned}$$

and using Eq. (27) its diffused concentration $c_{\mathrm{drug}}$ at position $\mathbf{r}$ with injected position $\mathbf{r}_d$ in desmoplastic ECM is expressed as,

28 $$\begin{aligned} \begin{aligned} c_{\mathrm{drug}}(\mathbf{r})&= \int _{0}^{t}\frac{{\gamma _{\mathrm{drug}}}(t)}{4\pi D_{\mathrm{drug}}(t-s)}e^{\frac{{\parallel \mathbf{r}}-\mathbf{r}_d\parallel ^2}{4D_{\mathrm{drug}}(t-s)}}\mathrm{d}s \\&= \int _{t_2}^{t_3}\frac{{\gamma _0}}{4\pi D_{\mathrm{drug}}(t-s)}e^{\frac{{\parallel \mathbf{r}}-\mathbf{r}_d\parallel ^2}{4D_{\mathrm{drug}}(t-s)}}\mathrm{d}s. \end{aligned} \end{aligned}$$

In pancreatic cancer, gemcitabine targets on inhibiting the proliferation of cancer cells. Moreover, this model can be extended to immunotherapy in the form of an injection of antibodies to boost the immune system or by the use of immune checkpoint inhibitors, etc. Cancer cells enable the immune checkpoint protein (like CTLA-4, PD-1, PD-L1, etc.) of T-lymphocytes to be over-expressed which is not conducive to the activation of T-lymphocytes.

To consider the variation in concentration of enzyme or drug, the circular desmoplastic ECM (see Fig. 1b) is divided into subdomains as shown in Fig. 2b. In each subdomain, the middle point is used to sense the enzyme/ drug concentration given by

29 $$\begin{aligned} \begin{aligned} \mathbf{x}_i=&\left( \frac{{R_1 + R_2}}{2} \cos (2\pi \frac{{(i-1)}}{\mathrm{N}}\right. ) ,\\&\frac{{R_1 + R_2}}{2} \sin \left. ( 2\pi \frac{{(i-1)}}{\mathrm{N}}) \right) ,\ \mathrm{for} \ i \in \{1,\ldots ,\mathrm{N}\}. \end{aligned} \end{aligned}$$

Here, $R_1$ and $R_2$ are the radii of the inner and outer boundaries, respectively, which are divided by $\mathrm{N} = 10$ points. We only use the subdomains during the treatments since the enzyme and drug concentrations exhibit variations over the periphery of the ECM around the tumor.

In experiments (Jacobetz et al. 2012), PEGPH20 treatment leads to a significant increase in fenestrae, interendothelial gaps and macromolecular permeability. Moreover, Shepard (2015) demonstrated that PEGPH20 treatment stimulates immune NK cells and trastuzumab penetration. Thereby, the antitumor response is boosted. The penetration of immune cells and macromolecular structure benefit from less HA in ECM, which additionally weakens the impact of desmoplastic ECM orientation on cell migration. In our previous study (Chen et al. 2018b), we used constants for eigenvalues $\lambda _1$ and $\lambda _2$ in Eq. (21) to calculate the desmoplastic ECM orientation. In the current work, $\lambda _2(t, \mathbf{r})$, denoting the tangential orientation component at position *r* in ECM, is adjusted over time by

30 $$\begin{aligned} \frac{{\partial \lambda _2(t, \mathbf{r}})}{\partial t} = \mathrm{L}(\lambda _1-\lambda _2(t, \mathbf{r}))c_{\mathrm{en}}(t, \mathbf{r}), \end{aligned}$$

where $\mathrm{L}$ is a rate constant. Let *n* be the time index, then subsequently $\lambda _2^{n+1}(\mathbf{r})$ can be approximated from the previous time step by

31 $$\begin{aligned} \lambda _2^{n+1}(\mathbf{r}) = \lambda _2^{n}(\mathbf{r}) + \mathrm{L}(\lambda _1-\lambda _2^{n}(\mathbf{r}))c^n_{\mathrm{en}}(\mathbf{r})\Delta t. \end{aligned}$$

Furthermore, the attenuation factor $e^{-ks}$ of radial velocity in Eq. (21) becomes time dependent as well, where $k(t,\mathbf{r})$ is changed to

32 $$\begin{aligned} \frac{{\partial k(t, \mathbf{r}})}{\partial t} = - \mathrm{L}k(t, \mathbf{r})c_{\mathrm{en}}(t, \mathbf{r}). \end{aligned}$$

Analogously, the $k^{n +1}(\mathbf{r})$ is updated by

33 $$\begin{aligned} k^{n+1}(\mathbf{r}) = k^{n}(\mathbf{r}) - \mathrm{L}k^{n}(\mathbf{r})c_{\mathrm{en}}^{n}(\mathbf{r})\Delta t. \end{aligned}$$

To locate each T-lymphocyte and to determine where they are at time *t* if they are in the desmoplastic ECM region, we compute the angle of the line segment between cell position and the center (0, 0) and the horizontal axis by

34 $$\begin{aligned} \begin{aligned}\theta _j &= {\left\{ \begin{array}{ll} \mathrm{atan}(\frac{y_j}{x_j}), \quad \mathrm{if} \ y_j \ge 0 \\ \pi +\mathrm{atan}(\frac{{y_j}}{x_j}), \quad \mathrm{if} \ y_j< 0 \end{array}\right. }\\ &\quad \mathrm{for} \ j \in {\mathbb{T}}(t), \quad \mathrm{if} \ R_1< \parallel \mathbf{r}_j\parallel < R_2 . \end{aligned} \end{aligned}$$

For each subdomain, the angle is $\frac{{2\pi }}{N}$ and the *j*-th subdomain has a range of angles given by

35 $$\begin{aligned} \theta _j \in \left[ (j-1)\cdot \frac{{2\pi }}{N}, \quad j\cdot \frac{{2\pi }}{N}\right] , \quad \mathrm{for} \ j \in \{1,...,\mathrm{N}\}. \end{aligned}$$

### Monte Carlo simulations

One of the advantages in our model is the efficiency in computational time, and therefore, we carry out Monte Carlo simulations to quantitatively investigate the propagation of uncertainties in the parameters. Parameters are sampled from a normal distribution $N(\mu , \ \sigma ^2)$, where $\mu$ and $\sigma$ denote the mean value and the standard deviation. The investigated variable $X \in \{F, D, \beta , k\}$ is given by

36 $$\begin{aligned} X \sim \mu + \sigma N(0, 1). \end{aligned}$$

Each simulation is terminated at 80 h or 150 h, and then, we investigate the final fraction of cancer cells $f_\mathrm{c}$ as an evaluation criteria for cancer development. Afterward, the sample correlation coefficient $\rho$ between variables and the final fraction of cancer cells $f_\mathrm{c}$ reads as

37 $$\begin{aligned} \rho =\frac{{\sum \limits _{j=1}}^{N_s}(X_j-{\bar{X}})(f_\mathrm{c}^j-{\bar{f}}_c)}{[\sum \limits _{j=1}^{N_s}(X_j-{\bar{X}})^2\sum \limits _{j=1}^{N_s} (f_\mathrm{c}^j-{\bar{f}}_c)^2]^{\frac{{1}}{2}}}. \end{aligned}$$

In Eq. (37), ${\bar{X}}$ and ${\bar{f}}_c$ represent the average values. Note that the linear sample correlation coefficient ranges in $[-1, \ 1]$.

## Numerical results

Since we have not yet access to clinical data, we estimate the input parameters based on the range of data provided in the references, which are listed in Table 1. Furthermore, we use mathematical intuition to approximate some of the parameters not available in the literature, e.g., diffusivity of enzyme PEGPH20, which is a kind of protein and its value refers to a study with a range of diffusion coefficients of proteins (Young et al. 1980). Moreover, the elasticity of T-lymphocytes is much bigger than the elasticity of epithelial cells, which results in a larger repulsive force if T-lymphocytes mechanically collide with other cells. Since variations in some parameters may have significant impact on the numerical results, Monte Carlo simulations are carried out to evaluate the uncertainties and correlations among variables, as well as the likelihood that the cancer develops up to a predescribed extent. For a couple of parameters, we use sampling from a normal distribution, see Table 2 for details.

**Table 1:** Input values

Parameters	Notation	Value and units	Source
Radius of cells	*R*	8 μm	De Paiva et al. (2006)
Radius of T-lymphocytes	$R_t$	5 μm	Estimated
Cell contraction force	*F*	30 kg μm/h^2^	Estimated
Substrate elasticity	$E_s$	5 kg/μm h^2^	Estimated
Cell elasticity	$E_c$	0.5 kg/μm h^2^	Estimated
Elasticity of T-lymphocytes	$E_t$	250 kg/μm h^2^	Estimated
Cell mobility coefficient	$\beta$	60 h^-1^	Estimated
Friction coefficient	$\upmu$	0.2	Vermolen and Gefen (2012)
Cytokine diffusivity	$D_\mathrm{c}$	5E3 μm^2^/h	Bookholt et al. (2016)
PEGPH20 diffusivity	$D_{\mathrm{en}}$	1E1 μm^2^/h	Young et al. (1980)
Drug diffusivity	$D_{\mathrm{drug}}$	1E4 μm^2^/h	Jeon et al. (2002)
Secretion rate	$\gamma$	5E6 mol/h μm^3^	Savinell et al. (1989)
Injection rate	$\gamma _0$	5E6 mol/h μm^3^	Estimated
Time step	$\mathrm{d}t$	0.01 $\mathrm{h}$	Estimated
Inner radius of T-islet	$R_1$	120 $\upmu \mathrm{m}$	Estimated
Outer radius of t-islet	$R_2$	200 $\upmu \mathrm{m}$	Estimated

**Table 2:** Mean and standard deviation in the Monte Carlo simulation sampling

Parameter	*F*	*D*	$\beta$	Inhibitor *k*
Value	(30, $3^2$)	(5000, $(500)^2$)	(60, $6^2$)	(0.3, $0.1^2$)

### T-islets with anisotropic desmoplastic ECM and Monte Carlo simulations

Pancreatic ductal adenocarcinoma is notorious for the extensive and stiff desmoplasia surrounding the tumor, which is thought to be rare in other types of cancers. Most studies have shown that this abnormal desmoplasia facilitates cancer initiation, survival and further metastasis (Salmon and Donnadieu 2012; Lachowski et al. 2017).

In our previous study (Chen et al. 2018b), we developed a cell-based model to describe the influence of anisotropic desmoplasia on the locomotion of T-lymphocytes. Due to the stiffness and anisotropy of the desmoplastic ECM, T-lymphocytes likely become trapped in the desmoplasia area and then preferably move in the direction of the fiber arrangement. Bougherara et al. (2015) demonstrated that the distribution and migration of T-lymphocytes rely on the density and orientation of collagen fibers.

**Fig. 3:**
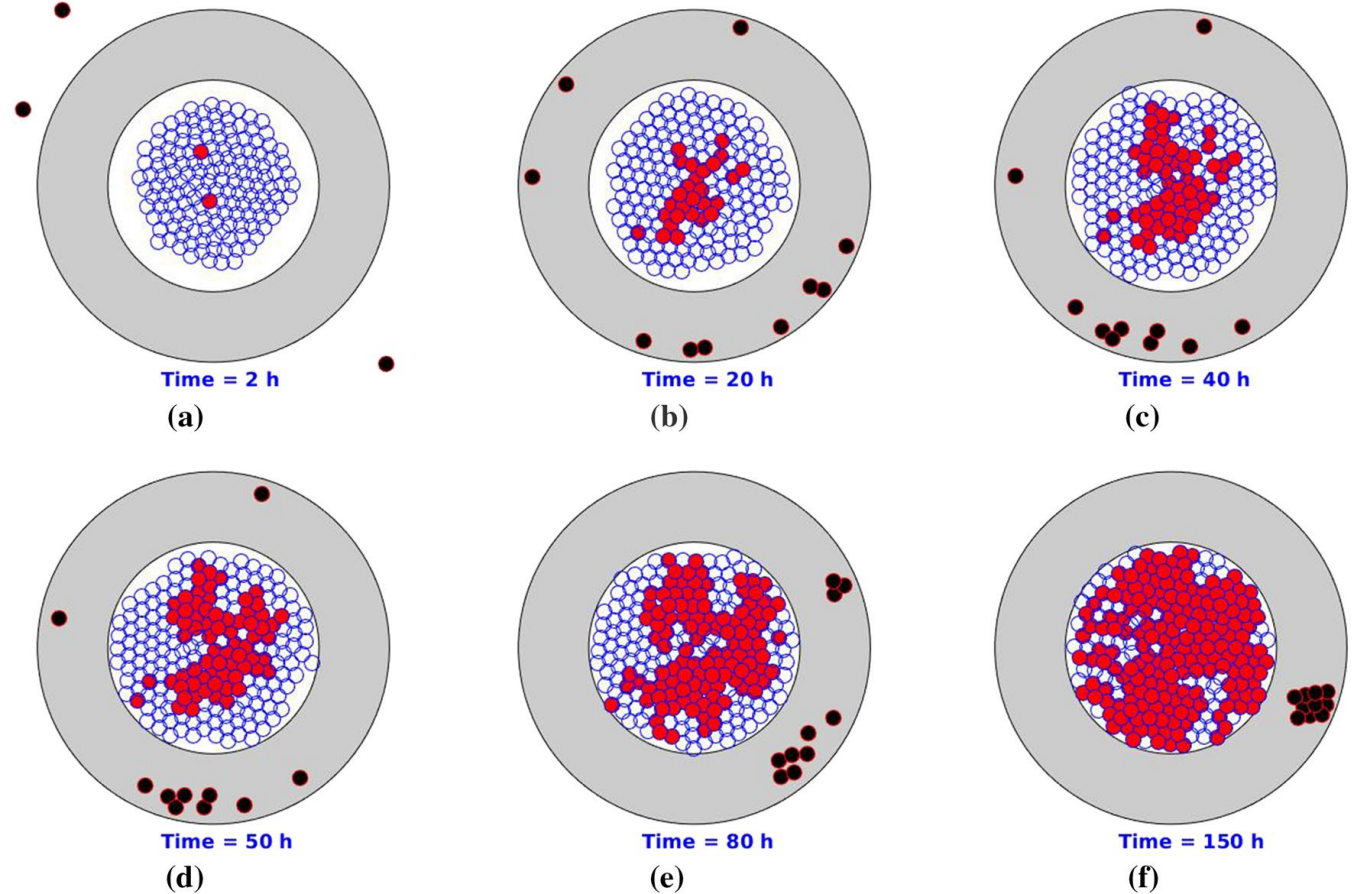
Snapshots of T-islets with desmoplastic ECM orientation. The epithelial cells, cancer cells, T-lymphocytes and anisotropic collagen are visualized by blue, red, black and gray colors, respectively

Since a chemotherapeutic drug administration cycle is typically one week, we restrict each simulation to 150 h. To make the problem tractable, we assume that the density of the collagen is uniform everywhere and that initially its arrangement is parallel to the T-islets circumference. Several consecutive snapshots of the numerical simulation are shown in Fig. 3, where epithelial cells, cancer cells, T-lymphocytes and anisotropic collagen are visualized by blue, red, black and gray colors, respectively. Due to the guide of the anisotropic orientation $\varPsi$, T-lymphocytes tend to accumulate in a certain area where the cancer cells secrete chemokine is maximal in the stromal layer. The tangential oriented ECM makes the T-lymphocytes unable to reach the cancer cells. As a result, the proportion of cancer cells of the total cells increases significantly within the T-islets. Our result is consistent with experimental observations in a study by Bougherara et al. (2015) on non-small cell lung cancer and ovarian cancer, where T-lymphocytes preferentially accumulate in the stroma rather than infiltrating into the cancer nest.

To investigate the influence of input parameters on the simulated results, Monte Carlo simulations are carried out, where input variables are sampled from statistical distributions, e.g., normal, uniform, Pareto, lognormal, exponential, etc (Mooney 1997). For an application of Monte Carlo simulations and error analysis, we refer to our cell deformation model (Chen et al. 2018a). To guarantee an acceptably small error, 5000 samples are used for the cell contraction force *F*, cytokine diffusivity $D_\mathrm{c}$, cell mobility coefficient $\beta$ and desmoplastic ECM inhibitor *k*. For the sake of saving computational time while ensuring that the results are not affected, we consider 80 h.

**Fig. 4:**
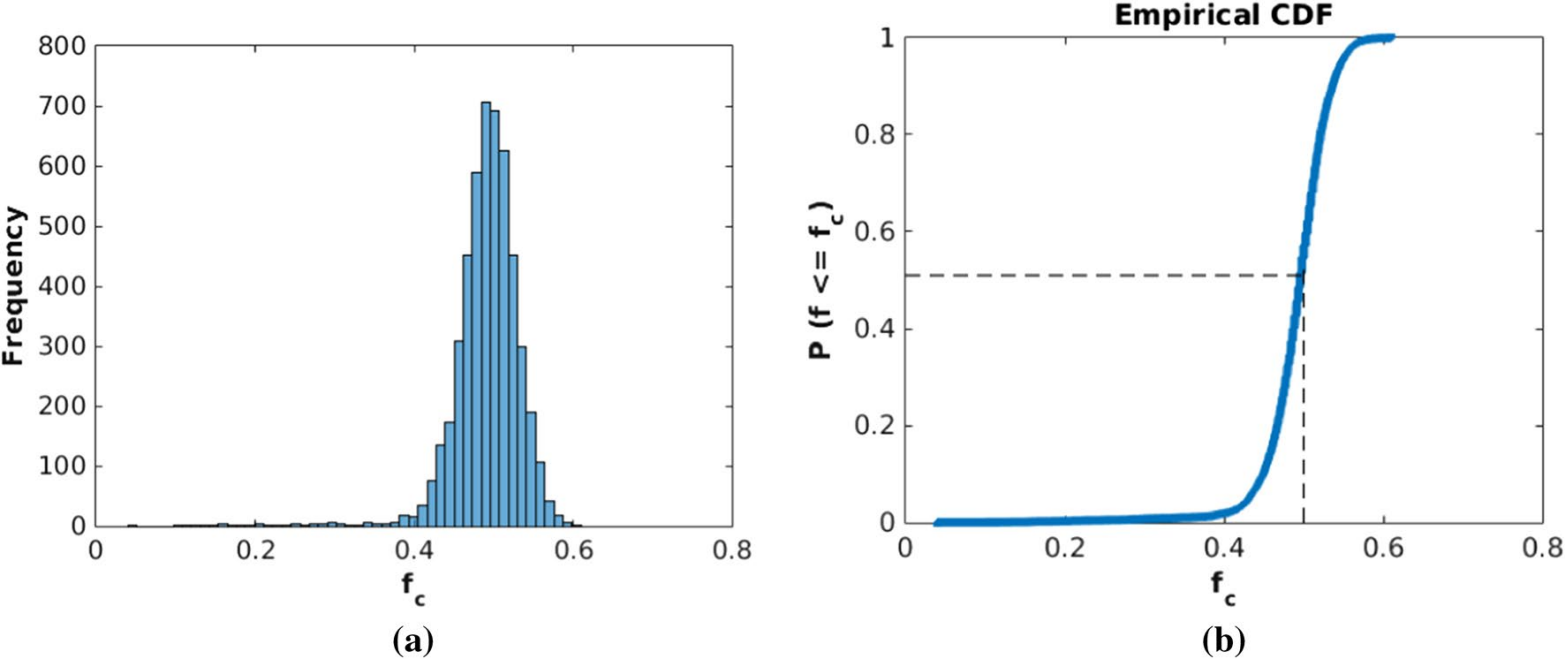
Histogram and CDF plot of outcomes at time of Monte Carlo simulations with 5000 samples. **a** There is no drug intervention. The x-axis shows the final fraction of cancer cells in total cells $f_\mathrm{c}$ and the y-axis is its corresponding frequency of occurrence. **b** Cumulative probability $f < f_\mathrm{c}$ based on the histogram in (**a**), where *f* is the dynamic fraction of cancer cells at time

In Fig. 4, we plot a histogram of 5000 samples for the fraction of cancer cells at the final time of the simulation $f_\mathrm{c}$ and a cumulative distribution function (CDF) of the estimated probability that $f_\mathrm{c}$ is lower than a certain number on the horizontal axis. As an example, the proportion of the cases where $f_\mathrm{c}$ is no more than 50% is approximately 51%. The probability rate of cell division, mutation and death in Eq. (17) is 100/h such that mutation happens during the interval of a time step in each simulation with a probability 0.63. For smaller probability rates for the mutation, we observed several cases in which no mutation, that is, no cancer, occurred. Moreover, this figure shows that most cases end with a large number of cancer cells as a result of ineffective T-lymphocytes infiltration.

**Fig. 5:**
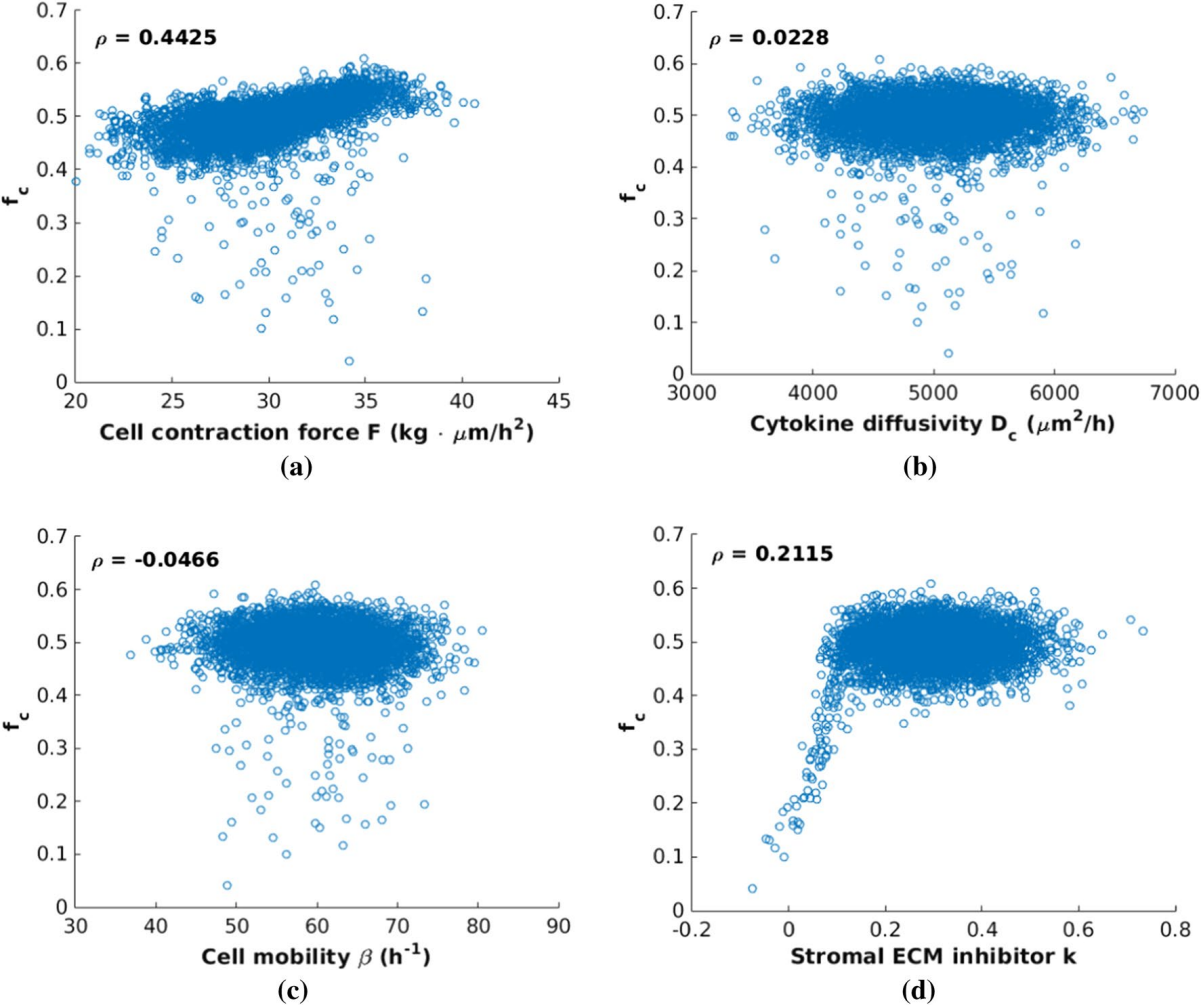
Scatter plots of the final fraction of cancer cells $f_\mathrm{c}$ at time = 80 h versus parameters *F*, $D_\mathrm{c}$, $\beta$ and *k*, respectively. The $\rho$ in each subfigure corresponds to its correlation coefficient. In comparison, **d** exhibits a significant correlation between $f_\mathrm{c}$ and the desmoplastic ECM inhibition faction *k* from 0 to 0.15

Subsequently, several scatter plots are listed in Fig. 5 showing the sample correlations between each input parameter with the estimated fraction of cancer cells $f_\mathrm{c}$. In comparison, significant impacts of the desmoplastic ECM inhibition *k* (in particular for small values) and cell contraction force *F* on $f_\mathrm{c}$ cannot be excluded, where other two variables, i.e., cytokine diffusivity $D_\mathrm{c}$ and cell mobility $\beta$, have no obvious correlations with $f_\mathrm{c}$. As expected, cancer cells grow and divide within the T-islets protected by the anisotropic desmoplastic ECM with influence from input parameters. The radical inhibition of the desmoplastic ECM becomes stronger as the desmoplastic ECM inhibition factor *k* increases and thereby the fraction $f_\mathrm{c}$ becomes relatively large. Note that there is a dramatic increase between 0 and 0.15, which means that the migration of T-lymphocytes is highly sensitive to the accumulation of HA, collagen, fibroblast, etc., in the early stages.

### PEGPH20 injection

Accumulated HA functions as a core polymer of the cancer-associated ECM to provide a hydrated viscoelastic gel-like matrix in collagenous fibers and forms the main barrier to chemotherapy delivery and vasculature (Thompson et al. 2010; Gore and Korc 2014). PEGPH20 is a type of enzyme aiming at depleting abundant HA in the anisotropic desmoplastic ECM to improve vascular perfusion and to increase effectiveness of anticancer therapeutics. Thompson et al. (2010) described their experiments, showing that PEGPH20 has an ability to remove the accumulated HA as well as to remodel the tumor microenvironment. Thence, we propose a simplified enzymatic depletion model of tumor stroma with PEGPH20 intervention to predict the interaction of cancer cells and its microenvironment.

**Fig. 6:**
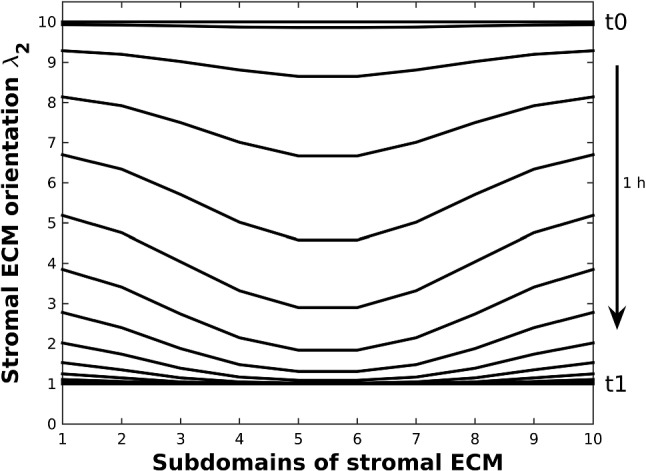
Variation of $\lambda _2$, which is the tangential component of desmoplastic ECM orientation, in ten subdomains (see Fig. 2b) during time interval $(t_0, t_1)$. Since the injection site of PEGPH20 is chosen at the middle of subdomain 5 and 6, but outside of the desmoplastic ECM, the orientation $\lambda _2$ of subdomain 5 and 6 decreases faster as a result of sensing a higher PEGPH20 concentration

To evaluate the variations in ECM orientation in different areas in Fig. 1b, the modeled anisotropic stroma is divided into ten subdomains in Fig. 2b. Each center of subdomains acts as a point to monitor the concentration of PEGPH20 that results in ten different concentration signals. Typically, PEGPH20 is given by intravenous injection in clinical trials while experimentally cell lines in vitro are fed with PEGPH20 in culture cell media. To develop a simplified model, we suppose that the injection site is just outside the T-islets near the subdomain 5. Normally, when patients have any symptoms, the pancreatic cancer is already in an advanced or late stages, which poses a challenge for the improvement in the prognosis. Since the model is developed for the early stage, we suppose that a high concentration of PEGPH20 is given when the number of cancer cells accounts for 35% of the total number marked as time $t_0$. The injection lasts 1 h to time $t_1$, and an attenuation of ECM orientation on T-lymphocytes migration within ten subdomains is shown in Fig. 6. The orientation degree $\lambda _2$ of subdomains is set to ten initially and subsequently decays during the time interval $(t_0,\ t_1)$, respectively, where the area near the injection site decays faster. Thereby, the T-lymphocytes in the PEGPH20-treated subdomains move faster in the radial direction at the beginning compared with in the rest subdomains. Note that eventually the orientation of the ECM has no significant influence on T-lymphocytes migration.

**Fig. 7:**
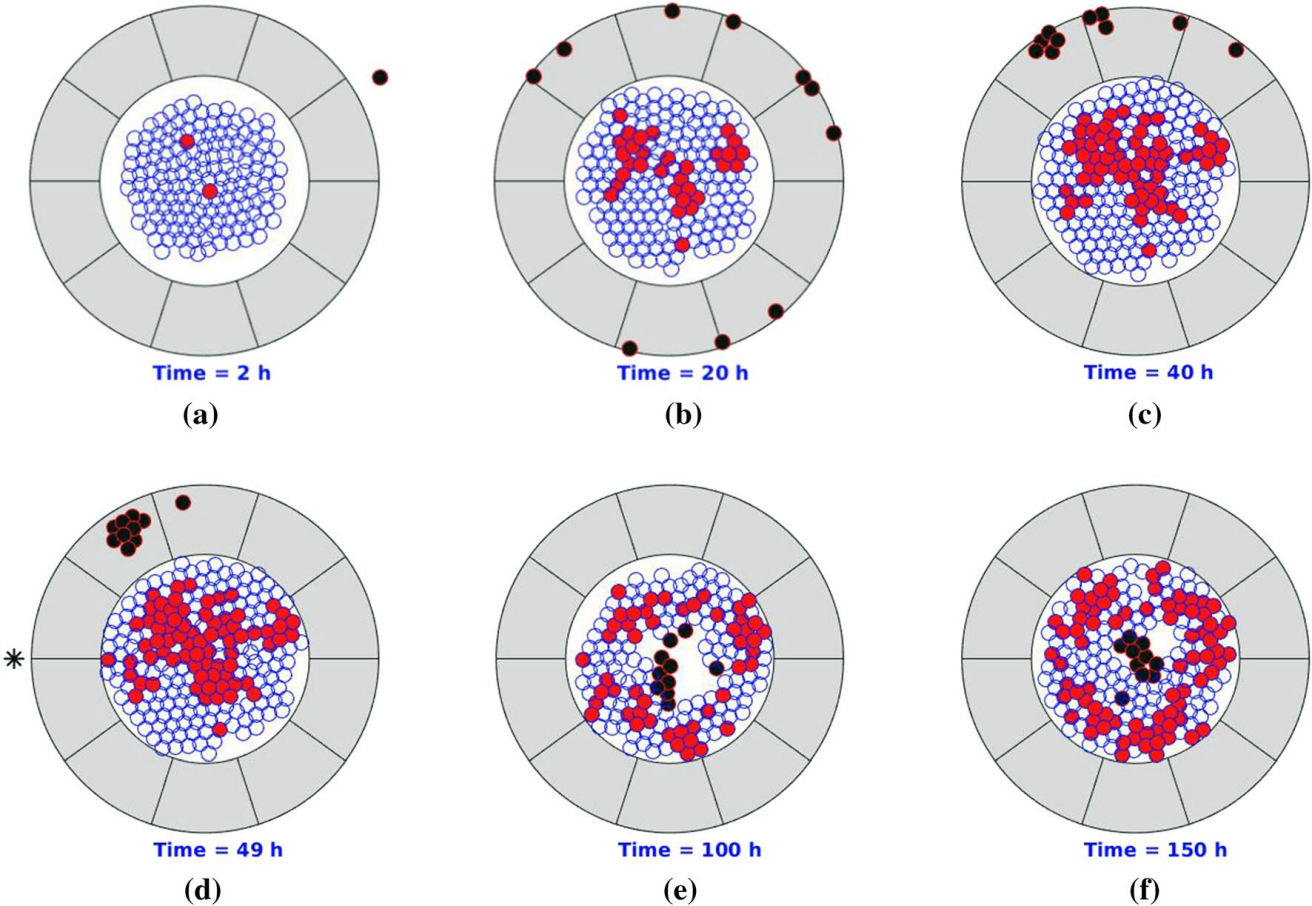
Snapshots of T-islets with PEGPH20 intervention. The epithelial cells, cancer cells, T-lymphocytes and anisotropic ECM are visualized by blue, red, black and gray colors, respectively. Moreover, the black asterisk is visualized as an injection site. Before PEGPH20 intervention, T-lymphocytes are trapped in peripheral ECM and accumulate in a certain area as a result of cancer-mediated chemotaxis and ECM orientation

Some consecutive snapshots are shown in Fig. 7 in which pancreatic cancer starts with epithelial cell mutation and triggers an immune response afterward. With ECM orientation, T-lymphocytes are trapped in peripheral ECM. After PEGPH20 is injected, T-lymphocytes are no longer hindered by the anisotropic desmoplastic ECM orientation in the solid stromal region and finally invade into the interior of the T-islets. However, the fraction of cancer cells remains stable in Fig. 7 when $t = 150$ h despite that the immune responses are boosted, since the cancer cells keep dividing without drug intervention. Therefore, drugs or antibodies are crucially important to fight uncontrolled cell division or to enhance the efficiency of the immune responses.

### PEGPH20 + gemcitabine injection

The aberrant desmoplasia is a result of activated pancreatic stellate cells which lead to production of collagen, laminin and fibronectin (Apte et al. 2013). As a consequence, cancer stroma exhibits abundant HA, increased stiffness and elevated hydrostatic pressure which collaborate to suppress the intratumoral drug delivery (Provenzano et al. 2012). With the enzymatic depletion of HA in the stromal region, the interstitial fluid pressure, which restores the vessel appearance and drug delivery into the carcinoma, decreases. Provenzano et al. (2012) experimentally studied the combinations of enzyme PEGPH20 and drug gemcitabine for the treatment of pancreatic cancer in the mice. To provide more predictions and possibilities, we develop a PEGPH20 + gemcitabine model for the treatment of pancreatic cancer.

**Fig. 8:**
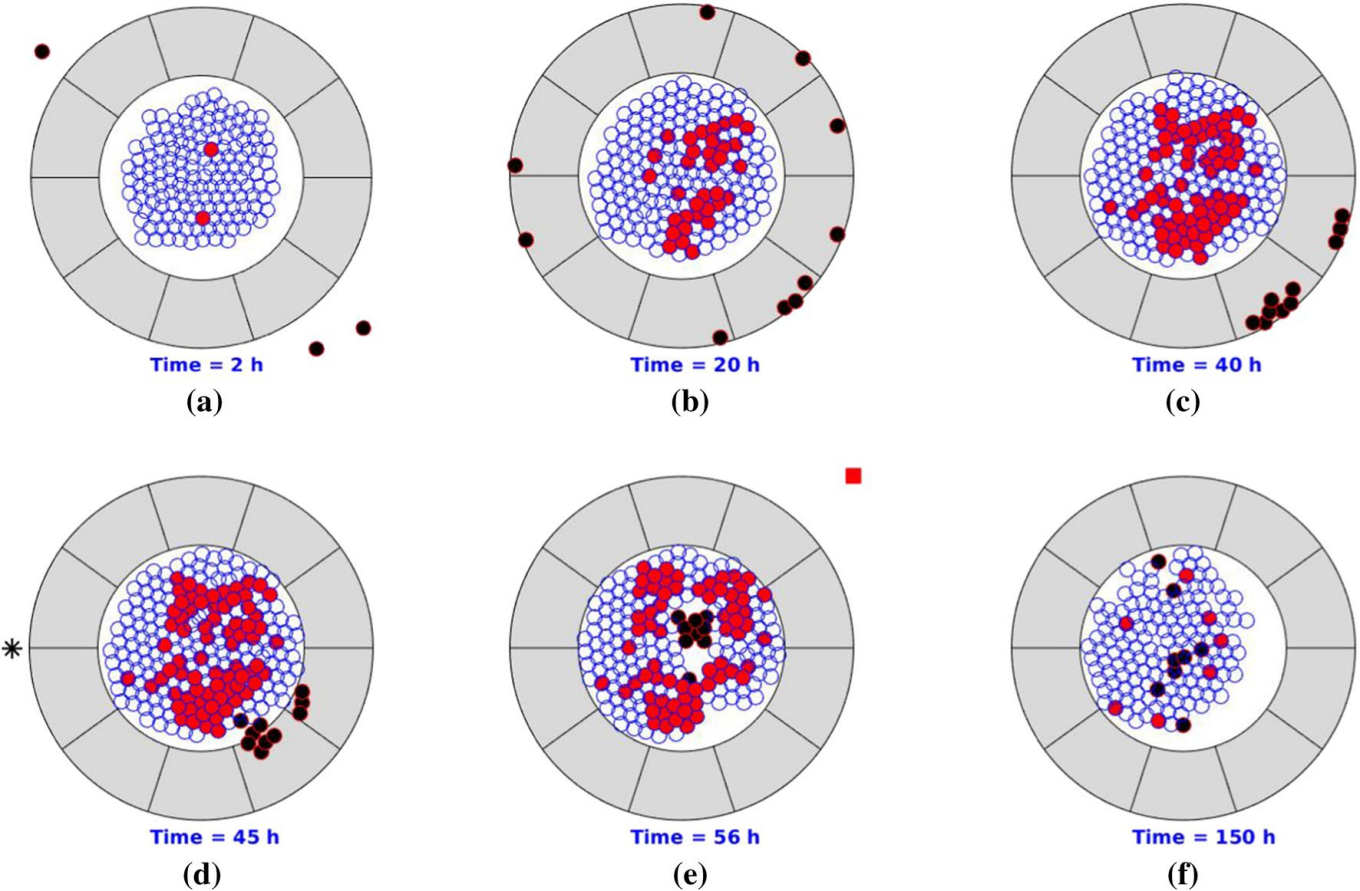
Snapshots of T-islets with intervention of PEGPH20 + gemcitabine. The epithelial cells, cancer cells, T-lymphocytes and anisotropic collagen are visualized by blue, red, black and gray colors, respectively. Moreover, the black asterisk is visualized as PEGPH20 injection site and the red-filled square denotes gemcitabine injection site. Before PEGPH20 intervention, T-lymphocytes are trapped in peripheral ECM and accumulate in a certain area as a result of cancer-mediated chemotaxis and ECM orientation

Gemcitabine is the first-line drug for pancreatic cancer, which inhibits processes required for DNA synthesis and causes cell death (Plunkett et al. 1995). In our earlier study (Chen et al. 2018b), the probabilistic division of cancer cells can happen under the following conditions: (1) sufficient time interval for growth and (2) suitable strain energy density. We assume that the probability rate during a time interval remains unchanged for cell mutation, division and death. Since the drug impedes cancer cell proliferation, we hypothesize that the probability rate $\lambda$ of cell mutation and cancer division depends on the concentration of gemcitabine $\lambda = \lambda (c(t))$. In the simulations, drug injection (indicated by a red-filled square) lasts 1 h such that the concentration of gemcitabine increases during an hour and subsequently goes down. Consequently, the probability of mutation and division of cancer decreases, and then, the T-lymphocytes are more likely to eliminate cancer cells. The resulting behaviors are shown by some snapshots in Fig. 8, where PEGPH20 is injected around $t = 45$ h. Due to the stochastic nature of the model, each simulation varies from others, and thereby, the injection time changes with $t = 49$ h in Fig. 7 and $t = 45$ h in Fig. 8. Therefore, we vary the fraction of cancer cells when injecting PEGPH20/ PEGPH20 + gemcitabine. The injection point is visualized as a black asterisk, and 10 h later (Jacobetz et al. 2012), gemcitabine is administered. Compared to Fig. 7, the final fraction of cancer cells $f_\mathrm{c}$ is much smaller in Fig. 8 at $t = 150$ h, which means that the combination of PEGPH20 + gemcitabine is more effective than the use of PEGPH20 only in order to facilitate concurrent immune response and chemotherapy.

**Fig. 9:**
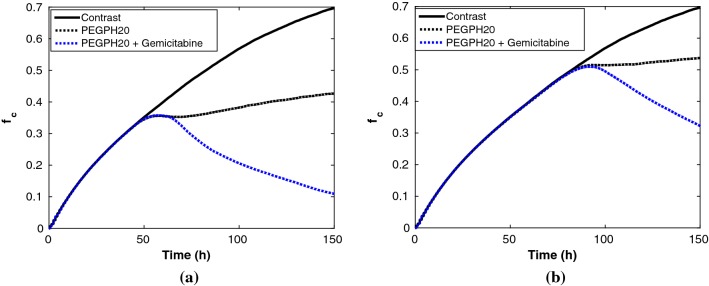
Comparisons of the final fraction of cancer cells $f_\mathrm{c}$ during 150 h evolution in T-islets for three cases. Three cases are: without treatment, with PEGPH20 alone and with PEGPH20 + gemcitabine, respectively. **a** Treatment starts as soon as the fraction of cancer cells is 35%. **b** Treatment starts as soon as the fraction of cancer cells is 50%

In animal-based experiments, the size of a solid pancreatic tumor has been compared before and after the combined treatment, respectively (Provenzano et al. 2012). We alternatively compare the fraction of cancer cells $f_\mathrm{c}$ in T-islets before and after treatment, for simplicity of calculation. Figure 9 shows the comparison of $f_\mathrm{c}$ in T-islets with PEGPH20 intervention only and with combined PEGPH20 + gemcitabine, respectively. Each simulation is restricted to 150 h, where PEGPH20 is given once; the initial cancer cell proportion is 35% and gemcitabine is injected 10 h later in the combined treatment referring to Jacobetz et al. (2012). In Fig. 9a, the number of cancer cells increases to the maximum capacity of the modeled T-islets (approximately 250 in the current simulation domain) because T-lymphocytes are trapped in the desmoplastic ECM area. With an early PEGPH20 intervention, T-lymphocytes are capable of penetrating the enzyme-depleted ECM to engulf cancer cells, and thereby, the fraction of cancer cells slightly drops firstly and then gradually rebounds into a growing trend toward roughly 42% in the end. Next, the combined PEGPH20 + gemcitabine is considered, as we expected, only a few cancer cells are finally left with a fraction of 11% after 150 h.

Aside from physical barriers, the influence of the injection time of PEGPH20/PEGPH20 + gemcitabine on the progression of cancer is crucially important. If the injection time is delayed until the cancer cells have accounted for 50% of the total cells, the corresponding result is shown in Fig. 9b. In terms of the final proportion of cancer cells $f_\mathrm{c}$, the PEGPH20 alone could restrict the fraction of cancer cells with a dynamic equilibrium for a short period. Furthermore, the follow-up progression of cancer depends on the patient’s own immune response, whereas PEGPH20 + gemcitabine could control the fraction of cancer cells to some extent due to functions of the drug. The model predicts roughly a fraction of 31% when $t = 150$ h, see Fig. 9b, where probably more PEGPH20 + gemcitabine is needed for the further treatment. During the cancer progression and therapy, the likelihood of cancer metastasis increases over time, and thereby, personalized therapeutic strategies are necessary, which can benefit from computational modeling.

**Fig. 10:**
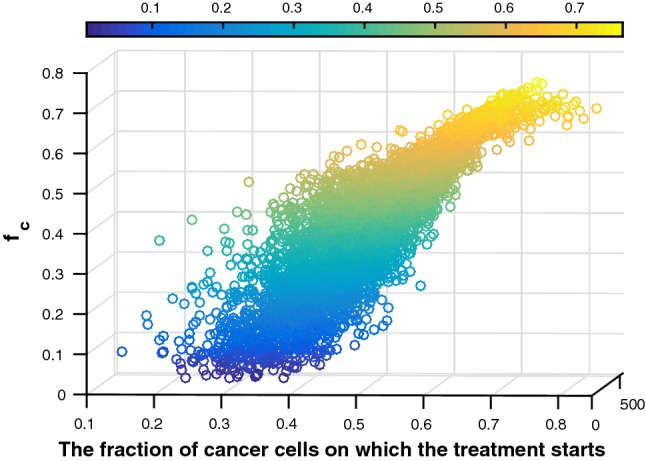
Three-dimensional scatter plot of fraction of cancer cells $f_\mathrm{c}$ at which the treatment is started, injection time interval and final fraction of cancer cells. The color bar indicates the final fraction of cancer cells. Blue colors indicate low final fractions of cancer cells, whereas yellow colors indicate high fractions of cancer cells. Hence, blue colors are favorable, whereas yellow colors are not

To investigate the fraction of cancer cells on which the combined treatment starts and the dose of the drug on the final fraction of cancer cells, Monte Carlo simulations are incorporated with 5000 samples. Note that a dose of the drug is calculated by $\gamma _{\mathrm{drug}} \times \tau$, where $\gamma _{\mathrm{drug}}$ is a constant injection rate and $\tau$ is a time interval. Therefore, the initial fraction of cancer cells when starting treatment as well as the time interval $\tau$ is sampled from a normal distribution with $(0.5, \ 0.1^2)$ and $(2, \ 1^2)$ h, respectively. The result in Fig. 10 shows a three-dimensional scatter plot of the initial fraction of cancer cells when starting treatment, injection time interval and the final fraction of cancer cells $f_\mathrm{c}$, where a horizontal color bar specifies the final fraction of cancer cells. If we aim that $f_\mathrm{c}$ does not exceed 20% in the pancreatic T-islets, the fraction of cancer cells at which the treatment is started when injection PEGPH20 + gemcitabine should never be larger than 40%. Hence, if this fraction exceeds 40%, then $f_\mathrm{c}$ will never be lower than 20% after 150 h. By applying Eq. (37), the sample correlation coefficient of fraction at which the treatment is started and $f_\mathrm{c}$ equals $\rho = 0.8785$. This result gives the prediction about the likelihood of a cure for specific patients after combining these two drugs. Probably, other therapies should be incorporated in if a patient is diagnosed at very late stages, or treatments should last longer.

**Fig. 11:**
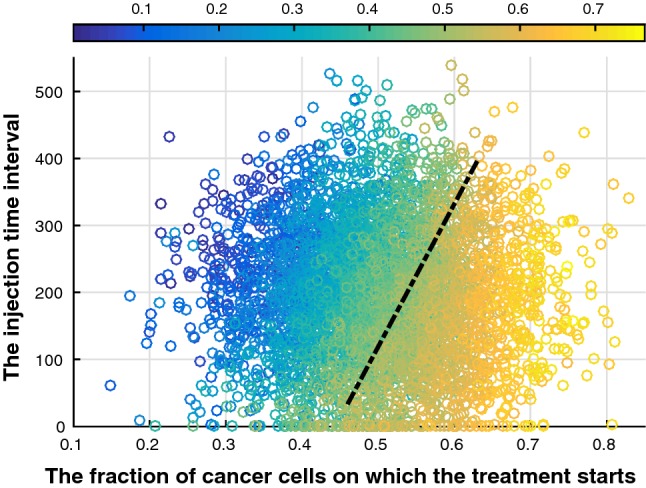
Scatter plot of the fraction of cancer cells on which the treatment starts, injection time interval and final fraction of cancer cells. Blue colors indicate low final fractions of cancer cells, whereas yellow colors indicate high fractions of cancer cells (see colorbar). Hence, blue colors are favorable, whereas yellow colors are not. A dashed black line is used to divide the potential consequences into two parts, where the likelihood of cure in the left side is higher, whereas the right side part hints a high risk of malignant cancer and even metastasis

Subsequently, Fig. 11 shows correlations between the injection time interval and the final fraction of cancer cells $f_\mathrm{c}$ under various treatment times. It hints that within our chosen range there is an obvious influence with a correlation coefficient $\rho = -\, 0.2753$ of doses of the drug on the final results. In other words, big doses of drugs are necessary if the treatment starts when the fraction of cancer cells exceeds 40%. Furthermore, the potential consequences are divided into two parts by a dashed line, where the likelihood of cure in the left side is higher, whereas the right side means a high risk of malignant cancer and probably metastasis. Taking the toxicity of the drug into consideration, large drug doses could be problematic for other parts of the body, and we think that this model is good for making choices of drug dosage.

## Conclusions and discussion

Pancreatic cancer is a lethal disease mainly due to late diagnosis, low resection rate, high recurrence, metastasis and chemotherapy resistance. Unfortunately, there is currently no standard programme for screening patients who have a high risk (Kamisawa et al. 2016). Combined with surgical resection, cytotoxic therapy plays an essential role in the standard treatment and in the prolongation of survival of pancreatic cancer. The frontline therapies normally involve the administering of gemcitabine combined with other drugs like cisplatin, epirubicin, 5-FU, etc. For a review of therapeutic strategies, we refer to Chiaravalli et al. (2017). However, the increased toxicity and various ethical concerns hinder the investigation and clinical administering of single and combined drugs. Mathematical modeling can shed light on the quantitative effects of drug combinations as well as provide reliable predictions.

We have developed a model for drug-oriented therapy of pancreatic cancer based on the simplification of the phenomenon and assumptions mentioned in Sect. 2. On the cellular level, our model is able to show the initial cancer progression and its interactions with the microenvironment. The normal epithelial cells are able to mutate into cancer cells under certain circumstances, which subsequently remodels the peripheral ECM and triggers T-lymphocytes-mediated immune response by secreting cytokines. In normal situations, the migration of the T-lymphocytes is guided by the desmoplastic ECM such that cancer cells in the T-islets can proliferate out of control because of lacking T-lymphocytes infiltration. After a PEGPH20 intervention, the enzyme-mediated degradation of ECM enhances T-lymphocytes penetration, and thereby, the cancer cells that are exposed to be T-lymphocytes are eliminated; however, this enzyme-mediated therapy is suitable for patients with an early diagnosis without immunodeficiency. For patients with advanced diagnosis, it is necessary to combine PEGPH20 with the drug gemcitabine, which is much more efficient for clearing the cancer cells. Additionally, this cell-based model could be upscaled to a large cell colony or even an organ scale, while the time at which the treatment starts, as well as the length of the time period of administration of different therapies can be personalized.

Furthermore, Monte Carlo simulations facilitate our model to investigate the uncertainties of input parameters and to predict the likelihood of a cure with various diagnosis stages. As a conclusion, the initial fraction of cancer cells when injecting the PEGPH20 has a significant sample correlation coefficient as high as 0.8785 with the final fraction of cancer cells. In contrast, the sufficient doses of drugs could reduce the final fraction of cancer cells in the current model with sample correlation coefficient − 0.2753. In summary, this therapy model is able to aid design the drug administering in the experiments. Further, the model can be extended to other therapy strategies like PEGPH20 + antibodies, PEGPH20 + cancer-targeted virus, PEGPH20 + cancer-targeted drugs, etc.

Albeit the computational models have their drawbacks like being too simplified, the mathematical modeling can be very helpful for the sake of prediction. For instance, Enderling et al. (2007) and Enderling et al. (2006) developed models of breast cancer that are beneficial for radiotherapy. Moreover, Tanaka et al. (2010) proposed a mathematical model which is helpful to prostate cancer therapy. On the other hand, animal-based experiments have moral concerns and systemic drugs normally have toxicity and strict restrictions regarding administering. Therefore, mathematical models can be used to optimize the drug therapies and further perform pre-validation studies before testing in animals or humans.

## Electronic supplementary material

Below is the link to the electronic supplementary material.

**S1** A model of T-islets without treatment. (MP4 5,352KB)

**S2** A model of T-islets with PEGPH20 treatment. (MP4 5,299KB)

**S3** A model of T-islets with PEGPH20 + gemcitabine treatment. (MP4 5,049KB)
